# Supplementary material for: Real‐world effectiveness of highly purified cannabidiol in epilepsy associated with 15q11.2‐q13.1 duplication and deletion syndromes: A multicenter study
Source: Epilepsia Open. 2026 Apr 16;11(3):1046–53. doi: 10.1002/epi4.70241 (PMC13238626; doi:10.1002/epi4.70241)
Supplement: Supplementary file 1 — Appendix [file EPI4-11-1046-s001.pdf]

Epilepsia Open®
The Open Access Journal of the International League Against Epilepsy

Real-world effectiveness of highly purified cannabidiol in epilepsy associated with 15q11.2-q13.1 duplication and deletion syndromes: a multicenter study

Journal:	*Epilepsia Open*
Manuscript ID	EPI4-0347-2025.R1
Wiley - Manuscript type:	Short Research Article
Date Submitted by the Author:	19-Dec-2025
Complete List of Authors:	Cerulli Irelli, Emanuele ; Universita degli Studi di Roma La Sapienza<br>Mazzeo, Adolfo; Universita degli Studi di Roma La Sapienza<br>Perulli, Marco; Fondazione Policlinico Universitario Agostino Gemelli IRCCS<br>Ramantani, Georgia; Universitats-Kinderspital Zurich Neuropadiatrie<br>Battaglia, Domenica Immacolata; Fondazione Policlinico Universitario Agostino Gemelli IRCCS; Universita Cattolica del Sacro Cuore - Campus di Roma<br>Bagnasco, Irene; Presidio Ospedaliero Martini<br>Cognolato, Erica; Presidio Ospedaliero Martini<br>Striano, Pasquale; Istituto Giannina Gaslini; Universita degli Studi di Genova Dipartimento di Neuroscienze Riabilitazione Oftalmologia Genetica e Scienze Materno-Infantili<br>Negrin, Susanna; Istituto di Ricovero e Cura a Carattere Scientifico Eugenio Medea Polo Scientifico di Conegliano-Pieve di Soligo<br>Danieli, Alberto; Istituto di Ricovero e Cura a Carattere Scientifico Eugenio Medea Polo Scientifico di Conegliano-Pieve di Soligo<br>Bonanni, Paolo; Istituto di Ricovero e Cura a Carattere Scientifico Eugenio Medea Polo Scientifico di Conegliano-Pieve di Soligo<br>Operto, Francesca; Universita degli Studi Magna Graecia di Catanzaro Scuola di Medicina e Chirurgia<br>Di Bonaventura, Carlo; Universita degli Studi di Roma La Sapienza<br>Coppola, Antonietta; Azienda Ospedaliera Universitaria Federico II<br>Orsini, Alessandro; Universita degli Studi di Pisa Dipartimento di Medicina Clinica e Sperimentale
Epilepsia Open Prize:	Yes
Topic:	Treatments: pharmacotherapy
Age Group:	Pediatric
Multicenter Research Submission:	No
Type of Research:	Clinical
Keywords:	CBD, developmental and epileptic encephalopathy, Lennox-Gastaut, Angelman syndrome, Dup15q syndrome

Junior Researcher Discount:	No
Abstract:	This multicenter retrospective study evaluated the effectiveness and safety of highly purified cannabidiol (CBD) in 22 patients with 15q11.2-q13.1 duplication or deletion syndromes (15q-DDS), including 12 with 15q duplication syndrome (dup15q) and 10 with Angelman syndrome (AS). Median (interquartile range [IQR]) age at CBD initiation was 14.5 (10-22.5) years, with a median (IQR) follow-up of 21 (14-33) months. All dup15q and two AS patients presented with a Lennox-Gastaut phenotype. At last observation, mean seizure reduction was 55.7% (95% confidence interval 38.7-72.7), with 63.6% patients achieving ≥50% reduction, 40.9% achieving ≥75% reduction, and 18.2% achieving seizure freedom. Tonic seizures in dup15q and myoclonic seizures in AS showed the most notable reductions. EEG improvement was observed in 7/16 patients, with marked improvement observed in two dup15q patients. Clinical improvement on the Clinical Global Impression-Improvement scale was reported in 72.7%, alongside non-seizure benefits such as improved sleep, behavior, and attention in a subset of patients. CBD was well tolerated; no patient discontinued CBD due to side effects alone, and retention at last visit was 81.8%. These findings suggest that CBD may provide clinically meaningful benefit in patients with 15q-DDS, including seizure reduction and improvements in sleep, behavior, and attention in selected cases.

SCHOLARONE™
Manuscripts

Real-world effectiveness of highly purified cannabidiol in epilepsy associated with 15q11.2-q13.1 duplication and deletion syndromes: a multicenter study

Emanuele Cerulli Irelli,[1*] Adolfo Mazzeo,[1] Marco Perulli,[2] Georgia Ramantani,[3] Domenica Battaglia,[2,4] Irene Bagnasco,[5] Erica Cognolato,[5] Pasquale Striano,[6,7] Susanna Negrin,[8] Alberto Danieli,[8] Paolo Bonanni,[8] Francesca F Operto,[9] Carlo Di Bonaventura,[1] Antonietta Coppola,[10**] Alessandro Orsini[11**]

* Corresponding author
** These authors equally contribute to this work

1 Department of Human Neurosciences, Sapienza University, Rome, Italy
2 Pediatric Neurology and Psychiatric Unit, Fondazione Policlinico Universitario Agostino Gemelli IRCCS, Rome, Italy
3 Department of Neuropediatrics, University Children's Hospital, Zurich, Switzerland
4 Department of Health Science and Public Health, Università Cattolica del Sacro Cuore.
5 Child Neuropsychiatry To-Sud Martini Hospital, Turin, Italy
6 Pediatric and Neuromuscular Diseases Unit, Member of the European Reference Network (ERN) for rare and complex epilepsies (EpiCARE), IRCCS Istituto Giannina Gaslini, Genoa, Italy
7 Department of Neurosciences, Rehabilitation, Ophthalmology, Genetics, Maternal and Child Health, University of Genoa, Genoa, Italy
8 Scientific Institute IRCCS E.Medea, Epilepsy and Clinical Neurophysiology Unit, Conegliano 31015, Italy
9 Department of Science of Health, School of Medicine, University of Catanzaro, Catanzaro, Italy
10 Neurology Unit; University Hospital Federico II, Naples, Italy
11 Department of Clinical and Experimental Medicine, University of Pisa, 56100 Pisa, Italy.

For Review Only

Number of words:
Abstract: 199
Manuscript body: 2440

Number of references: 20
Number of tables: 1
Number of figures: 1

Keywords: intellectual disability; CBD; developmental and epileptic encephalopathy; Lennox-Gastaut; Angelman syndrome; dup15q syndrome.

Corresponding author
Emanuele Cerulli Irelli, MD, PhD
Department of Human Neurosciences
Sapienza University, Rome, Italy
Viale dell'Università 30, 00185, Rome, Italy
Email: emanuele.cerulliirelli@uniroma1.it

Abstract

This multicenter retrospective study evaluated the effectiveness and safety of highly purified cannabidiol (CBD) in 22 patients with 15q11.2-q13.1 duplication or deletion syndromes (15q-DDS), including 12 with 15q duplication syndrome (dup15q) and 10 with Angelman syndrome (AS). Median (interquartile range [IQR]) age at CBD initiation was 14.5 (10-22.5) years, with a median (IQR) follow-up of 21 (14-33) months. All dup15q and two AS patients presented with a Lennox-Gastaut phenotype. At last observation, mean seizure reduction was 55.7% (95% confidence interval 38.7-72.7), with 63.6% patients achieving ≥50% reduction, 40.9% achieving ≥75% reduction, and 18.2% achieving seizure freedom. Tonic seizures in dup15q and myoclonic seizures in AS showed the most notable reductions. EEG improvement was observed in 7/16 patients, with marked improvement observed in two dup15q patients. Clinical improvement on the Clinical Global Impression-Improvement scale was reported in 72.7%, alongside non-seizure benefits such as improved sleep, behavior, and attention in a subset of patients. CBD was well tolerated; no patient discontinued CBD due to side effects alone, and retention at last visit was 81.8%. These findings suggest that CBD may provide clinically meaningful benefit in patients with 15q-DDS, including seizure reduction and improvements in sleep, behavior, and attention in selected cases.

Plain Summary

Epilepsy secondary to 15q11.2–13.1 duplication or deletion syndromes (15q-DDS) is often severe, making daily life difficult for patients and their families. In this study, treatment with highly purified cannabidiol (CBD) reduced seizures in many patients with 15q-DDS.
CBD was generally well tolerated and caregivers also reported improvements in sleep, behavior, and attention in a number of cases.
Overall, these findings suggest that CBD may be a helpful treatment option for people with 15q-DDS.

Key points

- Epilepsy associated with Angelman syndrome and dup15q is often severe and highly drug resistant.
- Evidence on the use of highly purified cannabidiol (CBD) in these genetic epilepsies has been limited to small case series and anecdotal reports.
- In this real-world study, add-on CBD was associated with a sustained reduction in seizure burden and a favorable safety profile.
- These findings provide a rationale for prospective studies evaluating CBD in these patient populations.

For Review Only

Introduction

Over the past few decades, the treatment landscape for epilepsy has significantly evolved, with an expanding armamentarium of anti-seizure medications (ASMs) available and a growing emphasis on personalized therapeutic approaches.[1]

Among recent advancements, highly purified cannabidiol (CBD) has been approved by the European Medicines Agency (EMA) and the U.S. Food and Drug Administration (FDA) for the treatment of drug-resistant epilepsy in Dravet syndrome (DS), Lennox-Gastaut syndrome (LGS), and tuberous sclerosis complex (TSC)-related epilepsy, based on evidence from randomized controlled trials.[2–4] Beyond these indications, emerging data suggest that CBD may also be effective in other developmental and epileptic encephalopathies (DEEs), especially those with an underlying genetic cause.[5,6]

Chromosome 15q syndromes are neurodevelopmental disorders caused by deletions or duplications within the unstable 15q11.2-q13.1 region, which is prone to clinically relevant rearrangements and contains genes subject to genomic imprinting.[7] Deletions in this region lead to either Prader-Willi syndrome (PWS) or Angelman syndrome (AS), depending on whether the deleted allele is of paternal or maternal origin. Conversely, maternally inherited duplications result in 15q duplication syndrome (dup15q), a clinically heterogeneous condition characterized by intellectual disability, autism spectrum disorder, hypotonia, and epilepsy in up to 80% of affected individuals, often presenting with a LGS phenotype.[8]

While epilepsy in PWS typically responds well to monotherapy with conventional ASMs,[9] both dup15q syndrome and AS are frequently associated with drug-resistant epilepsy.[7,10,11]

For Review Only

A previous open-label study suggested a possible effectiveness of CBD in patients with highly refractory epilepsy associated with dup15q.[12] Additionally, recent preclinical and anecdotal reports have highlighted a potential role for CBD in AS.[13,14]

With this background, we aimed to evaluate the effectiveness and safety of CBD in a cohort of patients with 15q11.2-q13.1 duplication / deletion syndromes (15q-DDS).

Methods

Data collection and inclusion criteria

This retrospective multicenter study was conducted across nine tertiary centers across Europe, with established expertise in epilepsy and DEEs, following STROBE guidelines. Written informed consent was obtained from the participants' legal guardians; assent was obtained from participants when appropriate.

The study was conducted in accordance with the Declaration of Helsinki and approved by the ethics committee of Sapienza University of Rome, Italy (protocol No. 7671, 0534/2024), with additional approvals obtained from local ethics committees in accordance with national regulations.

We included patients with drug-resistant epilepsy prescribed with CBD (Epidyolex®) from January 2020 to February 2024, who had a diagnosis of 15q-DDS, classified using established clinical and molecular diagnostic criteria.[7,15] Patients with <6 months of follow-up after CBD initiation were excluded.

Data were extracted retrospectively from medical records and included demographic characteristics, genetic findings, clinical history, neurodevelopmental comorbidities, seizure and epilepsy types, ASM history, and baseline seizure frequency, defined as the average monthly seizure count during the three months prior to CBD initiation. A shared, standardised data-collection sheet was used across centres to reduce variability in data extraction. Furthermore, when available, clinicians were asked to qualitatively evaluate the EEG tracings of the patients included in the study to assess whether any changes were observed over time.

For Review Only

Follow-up data on seizure frequency, according to seizure type, adverse events, and treatment discontinuation were obtained from seizure diaries, caregivers' reports and clinical records, with evaluations typically conducted at three-month intervals.[6]

CBD dosage and concomitant ASMs were adjusted by the treating clinicians during the follow-up as clinically indicated.

Effectiveness outcomes included mean seizure reduction, ≥50% and ≥75% seizure reduction rates, and seizure freedom at the last follow-up compared to baseline observation period. Safety and tolerability assessments included treatment retention and incidence of adverse events attributed to CBD by treating physicians.

Overall clinical response was also evaluated using the Clinical Global Impression–improvement (CGI-I) scale[16], a 7-point scale that ranges from 1 ('very much improved') to 7 ('very much worse'), which was applied retrospectively by the treating clinicians to assess perceived changes in efficacy and tolerability relative to baseline, incorporating the clinical status and the subjective report of the caregivers.

Furthermore, potential changes in domains not directly related to seizure outcomes—such as behaviour, sleep, alertness, or daily functioning—reported by caregivers or treating clinicians were also collected.

Statistical analysis

Based on data distribution and visual inspection, continuous variables were compared between groups using either an unpaired t-test (for overall seizure reduction) or the Mann–Whitney U test (for all other continuous variables), while Fisher's exact test was applied to categorical variables. Spearman's rank correlation was used to evaluate the association between percentage seizure reduction and CBD dose at the last follow-up.

CBD retention was assessed using survival analysis. The time of entry into the analysis was the date of CBD prescription, and the time of the endpoint was the date in which CBD was discontinued for

For Review Only

ineffectiveness or side effects or the date of the last follow-up visit (depending on which occurred first). A two-tailed p value < 0.05 was considered statistically significant.

Results

Clinical and demographic characteristics of patients

Twenty-two patients (10 females) with 15q-DDS were included, including 12 patients with dup15q syndrome and 10 patients with AS, with a median age at CBD initiation of 14.5 years (IQR: 10-22.5), All patients with dup15q syndrome and 2/10 (20%) with AS presented with an LGS phenotype. The most common seizure type in the overall population prior to CBD initiation was tonic in 13/22 (59.1%), followed by myoclonic in 9/22 (40.9%), atypical absences in 6/22 (27.3%), and spasms in 5/22 (22.7%).

At baseline, the study population was characterised by a high seizure burden and considerable prior treatment exposure. Most patients experienced daily seizures (15/22, 68.2%), while 5 patients (22.7%) had weekly seizures and only 2 patients (9.1%) had monthly or yearly seizures. At the time of CBD initiation, patients had been treated with a median of 5 antiseizure medications (IQR: 4–9.5). In addition, 5 patients (22.7%) had previous or concomitant treatment with vagus nerve stimulation, and 3 patients (13.6%) were receiving a ketogenic diet at the time of CBD initiation.

Detailed clinical and demographic characteristics of patients, as well as stratified data by diagnostic subgroup, are presented in Table. Detailed clinical and genetic data for individual patients are provided in the eTable in the Appendix.

Effectiveness of CBD

Median follow-up duration after CBD initiation was 21 months (IQR 14-33). The median initial maintenance dose of CBD was 10 mg/kg/day (IQR: 10–14), whereas the median dose at the last follow-up was 14.5 mg/kg/day (IQR: 11–18).

For Review Only

At last follow-up visit, mean seizure reduction was 55.7% (95% confidence interval [CI]: 38.7-72.7) and 14/22 (63.6%, 95% CI 43.0–80.3) patients achieved ≥50% seizure reduction, 9/22 (40.9%, 95% CI 23.3–61.3) achieved ≥75% seizure reduction, and 4/22 (18.2%, 95% CI 7.3–38.5) seizure freedom. No significant differences were noted regarding these effectiveness outcomes between AS and dup15q patients at any follow-up visit (p value >0.2 for all comparisons, Figure, panel A-B).

There was no statistically significant correlation between the final CBD dose and percentage seizure reduction at last follow-up (Spearman's Rho = –0.26, p = 0.24).

When considering the most common seizure types observed in our cohort, median reduction from baseline after CBD initiation was 60% (IQR 15-96) for tonic, 70% (IQR 0-75) for myoclonic, 50% (IQR 10-82) for atypical absences and 0% (IQR 0-25) for spasms.

Regarding concomitant ASM therapy, the regimen remained unchanged in 15 of 22 patients (68.2%). In five patients (22.7%), one ASM was withdrawn after CBD initiation (patients 1, 4, 5, 15, and 19). Conversely, in two patients, treatment adjustments involved ASM substitution, with phenytoin switched to cenobamate (patient 2) and brivaracetam switched to ethosuximide (patient 18).

The concomitant use of clobazam was not associated with a higher likelihood of achieving ≥50% and ≥75% seizure reduction or seizure freedom (p value >0.2 for all comparisons).

In patients with available pre- and post-treatment EEG tracings, a qualitative assessment showed a reduction in interictal epileptiform activity in 7/16 cases (43.7%). Complete disappearance of epileptiform discharges was observed in two patients with dup15q syndrome, one of whom also a showed marked improvement in background activity. In the eFigure, two representative examples are shown.

Safety, tolerability and overall efficacy of CBD

Regarding the CGI-I scale, applied retrospectively by the treating clinician, improvement was reported in 16/22 cases (72.7%, 95% CI: 51.8–86.8), with a significant association between CGI-I scores and overall seizure reduction at the last visit (Spearman's Rho = -0.561, p=0.007). Specifically, 4/16 (25%) patients were rated as very much improved, 5/16 (31.2%) as much improved, and 7/16

For Review Only

(43.7%) as slightly improved. No change was reported in 4/22 patients (18.2%), while worsening was noted in 2 (9.1%). Both cases of CGI-I worsening occurred in individuals with AS, with clinicians reporting behavioural worsening as the primary reason for the negative evaluation; in one case, this was associated with an increased intensity of myoclonic seizures. See panel C of the figure for CGI-I scores stratified by AS and dup15q.

Concerning non-seizure outcomes, caregivers reported improvements in sleep quality in 9/22 patients (40.9%), behavioral symptoms in 6/22 (27.3%), motor coordination and/or gait stability in 4/22 (18.2%), and overall attention and reactivity in 5/22 (22.7%). No changes across any domains were reported in 6/22 (27.3%), and behavioral worsening was noted in two, as previously described. See the eTable in the Appendix for individual-level data on non-seizure outcomes.

At the last visit, CBD retention was observed in 18/22 (81.8%, 95% CI: 61.5–92.7) patients (Figure, Panel D), with four patients (3 dup15q and one AS) discontinuing treatment due to lack of effectiveness, with no significant difference between the two groups (p value = 0.47).

Side effects were reported in six patients (27.3%, 95% CI 13.2–48.2), with only two patients still reporting them at the last visit. The most common side effects were drowsiness/sedation (5/22), followed by gastrointestinal complaints (3/22), elevated liver enzymes (1/22) and dermatological manifestations (1/22).

Among patients experiencing side effects, the median initial maintenance dose of CBD (12.5 mg/kg/day, IQR: 6.3-19.8) and the median dose at last follow-up (14.5 mg/kg/day, 6.2-19.5) were comparable to those in patients without side effects (10 mg/kg/day, IQR 10-11, and 11.4 mg/kg/day IQR 14.5-18.3, respectively), with no statistically significant differences between groups (p value > 0.2 for both comparisons).

Discussion

This is the largest real-world cohort study to date evaluating the effectiveness and tolerability of CBD in patients with 15q-DDS, encompassing both AS and dup15q syndrome. Before this work, evidence

For Review Only

was limited to a single case report in AS and a small dup15q subgroup within larger DEE cohorts, providing only preliminary indications of potential benefit from CBD treatment.[12,13]

In this cohort of 22 individuals with early-onset, drug-resistant epilepsy, CBD was associated with a clinically meaningful reduction in seizure burden. Mean seizure reduction reached 55.7% at last follow-up, with nearly two-thirds of patients achieving ≥50% reduction and almost 20% attaining seizure freedom. Importantly, the antiseizure effect was sustained over a median follow-up of nearly two years, with comparable outcomes in both AS and dup15q subgroups.

In particular, patients with dup15q syndrome presenting with an LGS phenotype showed a robust antiseizure effect, especially for tonic seizures. However, it is worth noting that a previous open-label expanded access program involving eight patients with dup15q syndrome, most of whom were treated during childhood, reported a lower responder rate.[12] This discrepancy highlights the need for further studies to identify clinical factors associated with response in this population.

For Review Only

Among patients with AS, apart from two individuals who were prescribed CBD due to an LGS phenotype, the majority were treated off-label given the occurrence of daily myoclonic seizures. A high response rate was reported, supporting the potential utility of CBD for the treatment of this seizure type in AS.

When considering CGI-I data, a well-established global measure of overall drug efficacy and tolerability[16], over 70% of patients were reported to experience clinical improvement, further supporting the antiseizure effect of CBD in this population. Beyond seizure reduction, non-seizure benefits such as improved sleep quality, gait stability, attention, and behavioral regulation were also observed in a subset of patients, suggesting potential broader neurobehavioral effects of CBD, consistent with findings in other genetic epilepsies.[6]

The pathophysiology of epilepsy in 15q-DDS is complex and not fully understood. The implicated chromosomal region includes more than 20 genes, among which *UBE3A*—a maternally expressed

imprinted gene involved in synaptic development and plasticity—and a cluster of non-imprinted *GABA_A* receptor subunit genes (*GABRB3*, *GABRA5*, *GABRG3*) are of particular interest. In dup15q, overexpression of UBE3A and these GABAergic genes may disrupt inhibitory tone, while in AS, haploinsufficiency leads to circuit hyperexcitability.[17]

Although limited by the small sample size of our cohort, it may be speculated that the ability of CBD to modulate GABAergic transmission—either by restoring inhibitory balance in dup15q or compensating for deficits in AS—may underlie its observed efficacy across both syndromes.[8,10,16,17] Future studies with pharmacogenomic markers may help clarify whether specific variants within GABA_A subunit genes or UBE3A predict differential CBD response.

Additionally, CBD is known to exert anxiolytic and antiseizure effects in part through activation of 5-HT1A receptors.[18] Recent findings from *Drosophila* models of dup15q syndrome possibly support a role for serotonergic modulation, showing that 5-HT1A agonists and 5-HT2B antagonists can act as potent seizure suppressors in this genetic disorder.[19] While these mechanistic hypotheses remain speculative, they provide interesting frameworks for understanding both the antiseizure and broader neurodevelopmental benefits observed in our cohort.

CBD was well tolerated. Retention at last follow-up was high (81.8%), and adverse effects were mild and consistent with previous studies, with no patient withdrawing treatment due to side effects in our cohort. Interestingly, concomitant clobazam treatment was not associated with a better outcome, in line with previous studies.[20]

Several limitations must be acknowledged. First, the retrospective and multicenter design of the study, although it allowed the inclusion of a considerable number of patients with such rare conditions, may have reduced homogeneity in data collection and introduced potential confounding. Furthermore, the relatively small sample size might limit the generalizability of our findings. Additionally, the assessment of seizure frequency during follow-up relied on caregiver reports,

For Review Only

seizure diaries, and clinicians' documentation, which may have introduced reporting bias. Likewise, non-seizure outcomes, CGI-I scale, and adverse events were derived from retrospective medical and caregiver reports rather than from standardized evaluations, potentially limiting the reliability of these measures. Finally, although improvements in EEG tracings were observed in a subset of patients, the assessment was limited to a qualitative evaluation.

In conclusion, our findings offer the most comprehensive real-world evidence to date on the effectiveness and tolerability of CBD in 15q-DDS, including both AS and dup15q. These results highlight the need for prospective studies exploring early treatment initiation, electrophysiological correlates, and mechanistic biomarkers. Given the substantial disease burden and drug resistance typical of epilepsy in 15q-DDS, CBD may emerge as a promising therapeutic option in these patients.

For Review Only

Figure. Effectiveness of cannabidiol in terms of mean seizure reduction (Panel A), responder rates and seizure freedom (Panel B), and caregiver-rated global clinical impression of change (Panel C), stratified by syndrome and in the overall population. Panel D shows CBD retention rate assessed by survival analysis.

For Review Only

We confirm that we have read the Journal's position on issues involved in ethical publication and affirm that this report is consistent with those guidelines.

Disclosures: E.C.I. received speaking honoraria from Angelini Pharma, outside the submitted work. A.M. received speaking honoraria and has served on advisory boards for Angelini Pharma, outside the submitted work. G.R. has served on advisory boards or received speaker honoraria paid to her department from Angelini, Bial, Jazz Pharmaceuticals, Neuraxpharm, Neurocrine, and Takeda, outside the submitted work. Her research receives support from the Swiss National Science (SNSF: 208184) and Anna Mueller Grocholski Foundations. D.B. has received consultancy honoraria from UCB-Pharma, Biocodex, Ethyl-Pharma and research support from Jazz Pharmaceuticals, outside the submitted work. P.S. has received speaker fees, consultancy honoraria, and/or research support from Jazz Pharmaceuticals UCB pharma, Angelini Pharma, Proveca, Biomarin, outside the submitted work. C.D.B. reports personal fees from UCB Pharma, Eisai, Jazz Pharmaceuticals, Angelini Pharma, Lusofarmaco, and Ecupharma, outside the submitted work. The other authors report no disclosures relevant to this article.

Author contributions: E.C.I., A.C. and A.O. contributed to the concept and design of the study. ECI contributed to drafting a significant portion of the manuscript and figures. All authors significantly contributed to the acquisition and analysis of the data and critically revised the manuscript for intellectual content.

Funding: None

Acknowledgments: None

Data availability: Anonymized data will be available to qualified academic investigators to replicate study results and as long as data transfer is in agreement with EU legislation on the general data protection regulation. Data transfer will be regulated by material transfer agreements and should be authorized by institutional Review Boards.

For Review Only

References

1. Knowles JK, Helbig I, Metcalf CS, et al. Precision medicine for genetic epilepsy on the horizon: Recent advances, present challenges, and suggestions for continued progress. *Epilepsia*. 2022;63(10):2461-2475. doi:10.1111/epi.17332
2. Devinsky O, Cross JH, Wright S. Trial of Cannabidiol for Drug-Resistant Seizures in the Dravet Syndrome. *N Engl J Med*. 2017;377(7):699-700. doi:10.1056/NEJMc1708349
3. Thiele EA, Bebin EM, Bhathal H, et al. Add-on Cannabidiol Treatment for Drug-Resistant Seizures in Tuberous Sclerosis Complex: A Placebo-Controlled Randomized Clinical Trial. *JAMA Neurol*. 2021;78(3):285-292. doi:10.1001/jamaneurol.2020.4607
4. Thiele EA, Marsh ED, French JA, et al. Cannabidiol in patients with seizures associated with Lennox-Gastaut syndrome (GWPCARE4): a randomised, double-blind, placebo-controlled phase 3 trial. *Lancet Lond Engl*. 2018;391(10125):1085-1096. doi:10.1016/S0140-6736(18)30136-3
5. Caraballo R, Reyes G, Demirdjian G, Huaman M, Gutierrez R. Long-term use of cannabidiol-enriched medical cannabis in a prospective cohort of children with drug-resistant developmental and epileptic encephalopathy. *Seizure*. 2022;95:56-63. doi:10.1016/j.seizure.2022.01.001
6. Cerulli Irelli E, Mazzeo A, Caraballo RH, et al. Expanding the therapeutic role of highly purified cannabidiol in monogenic epilepsies: A multicenter real-world study. *Epilepsia*. Published online March 24, 2025. doi:10.1111/epi.18378
7. Kalsner L, Chamberlain SJ. Prader-Willi, Angelman, and 15q11-q13 duplication syndromes. *Pediatr Clin North Am*. 2015;62(3):587-606. doi:10.1016/j.pcl.2015.03.004
8. Battaglia A. The inv dup (15) or idic (15) syndrome (Tetrasomy 15q). *Orphanet J Rare Dis*. 2008;3:30. doi:10.1186/1750-1172-3-30
9. Verrotti A, Soldani C, Laino D, d'Alonzo R, Grosso S. Epilepsy in Prader-Willi syndrome: clinical, diagnostic and treatment aspects. *World J Pediatr WJP*. 2014;10(2):108-113. doi:10.1007/s12519-014-0478-9
10. Battaglia A, Bernardini L, Torrente I, Novelli A, Scarselli G. Spectrum of epilepsy and electroencephalogram patterns in idic (15) syndrome. *Am J Med Genet A*. 2016;170(10):2531-2539. doi:10.1002/ajmg.a.37844
11. Samanta D. Epilepsy in Angelman syndrome: A scoping review. *Brain Dev*. 2021;43(1):32-44. doi:10.1016/j.braindev.2020.08.014
12. Devinsky O, Verducci C, Thiele EA, et al. Open-label use of highly purified CBD (Epidiolex®) in patients with CDKL5 deficiency disorder and Aicardi, Dup15q, and Doose syndromes. *Epilepsy Behav EB*. 2018;86:131-137. doi:10.1016/j.yebeh.2018.05.013
13. Pietrafusa N, De Palma L, Armando M, Corsetti T, Vigevano F, Specchio N. Successful use of cannabidiol in nonconvulsive status epilepticus in Angelman syndrome. *Epilepsia Open*. 2024;9(5):1997-1999. doi:10.1002/epi4.12948
14. Gu B, Zhu M, Glass MR, et al. Cannabidiol attenuates seizures and EEG abnormalities in Angelman syndrome model mice. *J Clin Invest*. 2019;129(12):5462-5467. doi:10.1172/JCI130419

For Review Only

15. Riggs ER, Andersen EF, Cherry AM, et al. Technical standards for the interpretation and reporting of constitutional copy number variants: a joint consensus recommendation of the American College of Medical Genetics and Genomics (ACMG) and the Clinical Genome Resource (ClinGen). *Genet Med Off J Am Coll Med Genet*. 2020;22(2):245-257. doi:10.1038/s41436-019-0686-8
16. Busner J, Targum SD. The clinical global impressions scale: applying a research tool in clinical practice. *Psychiatry Edgmont Pa Townsh*. 2007;4(7):28-37.
17. Reiter LT. Chapter 9 - Developmental disabilities, autism, and schizophrenia at a single locus: complex gene regulation and genomic instability of 15q11-q13 cause a range of neurodevelopmental disorders. In: Rubenstein J, Rakic P, Chen B, Kwan KY, eds. *Neurodevelopmental Disorders*. Academic Press; 2020:201-221. doi:10.1016/B978-0-12-814409-1.00009-4
18. Martínez-Aguirre C, Carmona-Cruz F, Velasco AL, et al. Cannabidiol Acts at 5-HT1A Receptors in the Human Brain: Relevance for Treating Temporal Lobe Epilepsy. *Front Behav Neurosci*. 2020;14:611278. doi:10.3389/fnbeh.2020.611278
19. Landaverde S, Sleep M, Lacoste A, et al. Glial expression of Drosophila UBE3A causes spontaneous seizures that can be modulated by 5-HT signaling. *Neurobiol Dis*. 2024;200:106651. doi:10.1016/j.nbd.2024.106651
20. Kühne F, Becker LL, Bast T, et al. Real-world data on cannabidiol treatment of various epilepsy subtypes: A retrospective, multicenter study. *Epilepsia Open*. 2023;8(2):360-370. doi:10.1002/epi4.12699

For Review Only

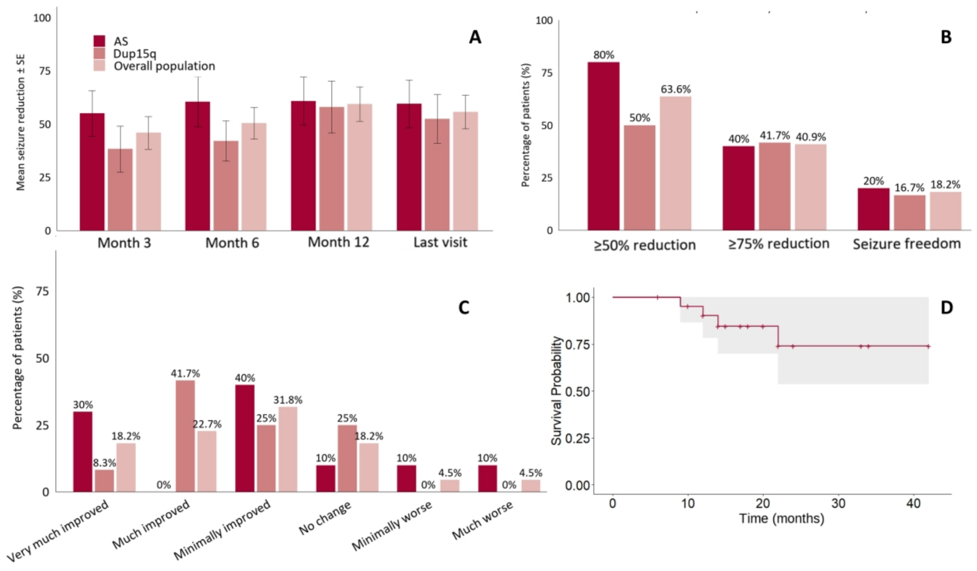

338x190mm (300 x 300 DPI)

**Table. Clinical characteristics of patients**			
	AS (10 pts)	Dup15q (12 patients)	Overall population (22 pts)
Demographic characteristics			
Female sex, n (%)	5 (50)	5 (41.7)	10 (45.5)
Intellectual disability severity			
Mild/moderate, n (%)	1 (10)	2 (16.7)	3 (13.6)
Severe, n (%)	9 (90)	10 (83.3)	19 (86.4)
Age at CBD initiation, median (IQR)	15.5 (6.5-27)	14.5 (10-17)	14.5 (10-22.5)
Follow-up duration, months, median (IQR)	16 (12-25.5)	23 (17-41)	21 (14-33)
Epilepsy characteristics			
Seizure type at CBD initiation			
Tonic seizures, n (%)	1 (10)	12 (100)	13 (59.1)
Myoclonic seizures, n (%)	8 (80)	1 (8.3)	9 (40.9)
Atypical absences, n (%)	1 (10)	5 (41.7)	6 (27.3)
Tonic-clonic seizures, n (%)	2 (20)	2 (16.7)	4 (18.2)
Atonic seizures, n (%)	1 (10)	1 (8.3)	2 (9.1)
Focal seizures, n (%)	2 (20)	2 (16.7)	2 (9.1)
Spasms, n (%)	1 (10)	4 (33.3)	5 (22.7)
Focal status epilepticus, n (%)	1 (10)	0	1 (4.5)
Frequency of seizure at baseline			
Daily, n (%)	7 (70)	8 (66.7)	15 (68.2)
Weekly, n (%)	1 (10)	4 (33.3)	5 (22.7)
Monthly/Yearly, n (%)	2 (20)	0	2 (9.1)
Number of ASM ever used at the time of CBD initiation, median (IQR)	5 (4.5-9.5)	5.5 (4-11)	5 (4-9.5)
Concomitant use of clobazam, n (%)	4 (40)	7 (58.3)	11 (50)
Abbreviations: ASM = anti-seizure medication; CBD = highly purified cannabidiol; IQR = interquartile range.			

For Review Only
